# Blockade of TRPV2 is a Novel Therapy for Cardiomyopathy in Muscular Dystrophy

**DOI:** 10.3390/ijms20163844

**Published:** 2019-08-07

**Authors:** Yuko Iwata, Tsuyoshi Matsumura

**Affiliations:** 1 Department of Clinical Research and Development, National Cerebral and Cardiovascular Center, 6-1 Kishibe Shinmachi, Suita, Osaka 564-8565, Japan; 2 Department of Neurology, National Hospital Organization Osaka Toneyama Medical Center, 5-1-1 Toneyama, Toyonaka, Osaka 560-8552, Japan

**Keywords:** TRPV2, muscular dystrophy, cardiomyopathy, heart failure, TRPV2 inhibitors

## Abstract

Muscular dystrophy and dilated cardiomyopathy are intractable diseases and their treatment options are very limited. Transient receptor potential cation channel subfamily V, member 2 (TRPV2), is a stretch-sensitive Ca^2+^-permeable channel that causes sustained intracellular Ca^2+^ increase in muscular cells, which is a pathophysiological feature of degenerative muscular disease. Recent reports have clarified that TRPV2 is concentrated and activated in the sarcolemma of cardiomyocytes/myocytes during cardiomyopathy/heart failure and muscular dystrophy. Furthermore, these reports showed that inactivation of TRPV2 ameliorates muscle dysgenesis to improve cardiac function and survival prognosis. Although TRPV2 is a potential therapeutic target for cardiomyopathy, there were no TRPV2 inhibitors available until recently. In this review, we introduce our recent findings and discuss the current progress in the development of TRPV2 inhibitors and their therapeutic applications for cardiomyopathy associated with muscular dystrophy.

## 1. Introduction

Muscular dystrophy (MD) and dilated cardiomyopathy (DCM) are intractable diseases and their treatment options are currently very limited. MD is characterized by degeneration and necrosis of the skeletal muscle resulting in progressive muscle weakness and atrophy. Diseases caused by mutations in a gene encoding cytoskeletal protein dystrophin are called dystrophinopathies, which include Duchenne muscular dystrophy (DMD), Becker muscular dystrophy (BMD), and DMD gene-related DCM. In addition to dystrophinopathies, many muscle diseases are caused by genetic abnormalities in proteins which form complexes with dystrophin (e.g., dystrophin–glycoprotein complexes [1]), and it has been reported that abnormalities in the same responsible genes as those of MD also cause DCM [2]. Some types of MDs exhibit a high incidence of combined myocardial and conduction dysfunction that leads to DCM. Mechanical ventilation has been widely used in patients with MD. Therefore, heart failure, rather than respiratory failure, has become an increasingly common cause of death in patients with MD. Elucidation of the onset mechanisms of MD and DCM has been in progress, but there are still lots of unknown parts. Although steroid treatment is a standard treatment for DMD, for other MDs there are few established treatments (treatments based on the evidence) as indications for medical insurance, and only some new drugs at the development stage. In these cases, drugs such as angiotensin-converting enzyme (ACE) inhibitors, angiotensin II receptor blockers (ARBs), beta-blockers, and aldosterone antagonists are prescribed as a standard therapy to treat heart failure in these patients. However, since many patients do not respond to these drugs, it is necessary to develop new therapeutic agents as soon as possible.

## 2. Muscular Dysgenesis and TRPV2

**Figure 1:**
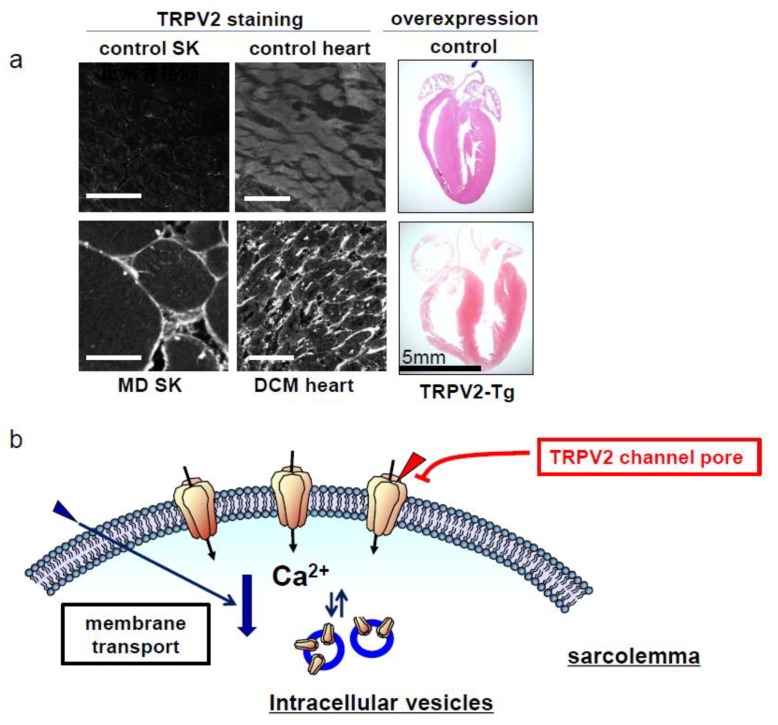
Membrane localization of TRPV2 channel in muscular dystrophy and cardiomyopathy. (**a**) Immunohistochemical localization of transient receptor potential cation channel subfamily V, member 2 (TRPV2) in frozen sections of skeletal muscles or cardiac muscles from the patients with muscular dystrophy (MD) and without MD (control) (from [8]), and the patients with dilated cardiomyopathy (DCM) and without DCM (control) (from [9]). Note the extensive sarcolemmal localization of TRPV2 in MD and DCM patients. Longitudinal sections of Masson’s trichrome staining hearts from the control and heart, specifically TRPV2 overexpressed in a transgenic (Tg) mouse. Scale bar = 100 μm. (**b**) A schematic drawing for the possible methods to inhibit the Ca^2+^ influx. TRPV2 channels localized in sarcolemma in muscle degenerative diseases can be blocked by stimulating internalization or inhibiting channel activity.

Disruption of the dystrophin–glycoprotein complex may significantly compromise the integrity of the cellular membrane and stability during muscle contraction/relaxation, which can lead to reduced survival of myocytes [3,4]. Enhanced susceptibility to muscle damage is observed in dystrophic animals, such as dystrophin-deficient *mdx* mice and δ-sarcoglycan (SG)-deficient J2N-k hamsters, which exhibit cardiac and skeletal abnormalities similar to those observed in human patients with DMD or limb-girdle muscular dystrophy. A chronic elevation in cytosolic Ca^2+^ concentration ([Ca^2+^]_i_) beneath the sarcolemma and within other cellular compartments has been reported in skeletal muscle fibers and cultured myotubes derived from DMD patients and *mdx* mice [5,6]. Previously, we have shown that δ-SG-deficient myotubes are highly susceptible to mechanical stretch and enhanced Ca^2+^ influx via the stretch-activated nonselective Ca^2+^ channel [7], and subsequently we identified transient receptor potential cation channel subfamily V, member 2 (TRPV2) as a membrane protein responsible for enhanced Ca^2+^ entry [8]. In recent years, we reported that TRPV2 accumulates in the sarcolemma of skeletal and heart muscle cells of patients with MD and DCM [8,9] (Figure 1a). Conversely, TRPV2 is localized to the intracellular compartments and intercalated discs of control cardiomyocytes [8,9].

A similar accumulation of TRVP2 in the sarcolemma was observed [9] in heart cells from cardiomyopathic J2N-k hamsters, *mdx* mice, and the murine models of DCM (sugar chain abnormal 4C30 DCM mice [10] and cardiac troponin T mutant knock-in mice (TNNT2 Δ210K) [11]). In addition, TRPV2 channel activity was enhanced in these cardiomyocytes, as evidenced by the high Ca^2+^-, 2-aminoethoxy diphenyl borate (2-APB) or stretch-induced increase in [Ca^2+^]_i_ [9]. Transgenic (Tg) mice that overexpress TRPV2 in cardiac muscle cell membranes also developed DCM due to Ca^2+^ overload- induced muscular degeneration [8] (Figure 1a).

Recent studies using rodent models of transverse aortic constriction (TAC)-induced heart failure [12], myocardial infarction (MI) [13,14], and chemotherapy-induced cardiomyopathy [9] showed that the enhanced TRPV2 expression was associated with cardiac dysfunction and that TRPV2 plays an important role in general heart failure and cardiomyopathy [15].

## 3. TRPV2 as a Drug Discovery Target

TRPV2 has three characteristics that make it an excellent therapeutic target. Firstly, TRPV2 is not present in the plasma membrane under normal conditions but translocates to the plasma membrane during disease states, where it contributes to excessive Ca^2+^ influx into the cells. Therefore, a drug targeting TRPV2 would be selective for dysfunctional cells, rather than normal cells. Secondly, there are two fundamental strategies for blocking TRPV2 signaling: Blocking its accumulation in the plasma membrane (stimulating internalization) or Ca^2+^ influx (Figure 1b). Thirdly, since [Ca^2+^]_i_ overload via TRPV2 activation is a common factor in the terminal phase of muscular degenerative diseases, TRPV2 inhibition may be effective in treating other dystrophinopathy-related diseases, even though their responsible gene mutations have not been identified yet.

We performed an experiment to test the safety of TRPV2 inhibition by administering TRPV2-neutralizing antibodies (over 2× the effective dose) to wild-type mice (WT) or isolated cardiomyocytes. We observed no obvious detrimental effects on both the behavior and physical parameters of the animals nor on the function of the cardiomyocytes (Iwata, Y et al., unpublished observation). It has been confirmed by many laboratories that the deletion of *TRPV2* in whole-body *TRPV2* knockout mice (*TRPV2* KO) does not cause any significant cardiac dysfunction or severe symptoms [16]. We also observed that the adult *TRPV2* KO mice (BL6/J) were not phenotypically different from the WT mice, and they have normal cardiac function. However, *TRPV2* KO is more likely to lead to embryonic lethality compared with WT mice (https://animal.nibiohn.go.jp/e_v2d.html) (the reason for the embryonic lethality of the BL6/J strain is still unknown).

## 4. Effects of Various TRPV2 Blockers on Muscle Dysgenesis and Cardiac Dysfunction

To confirm that TRPV2 is the therapeutic target for cardiomyopathy/heart failure and MD, it is important to determine whether heart failure and muscular dysfunction could be ameliorated by the specific inhibition of TRPV2 activity. Several ways to inhibit TRPV2 have been investigated using various animal models, which can be found in Table 1.

### 4.1. Amino Terminal (NT) Domain Expression

**Figure 2:**
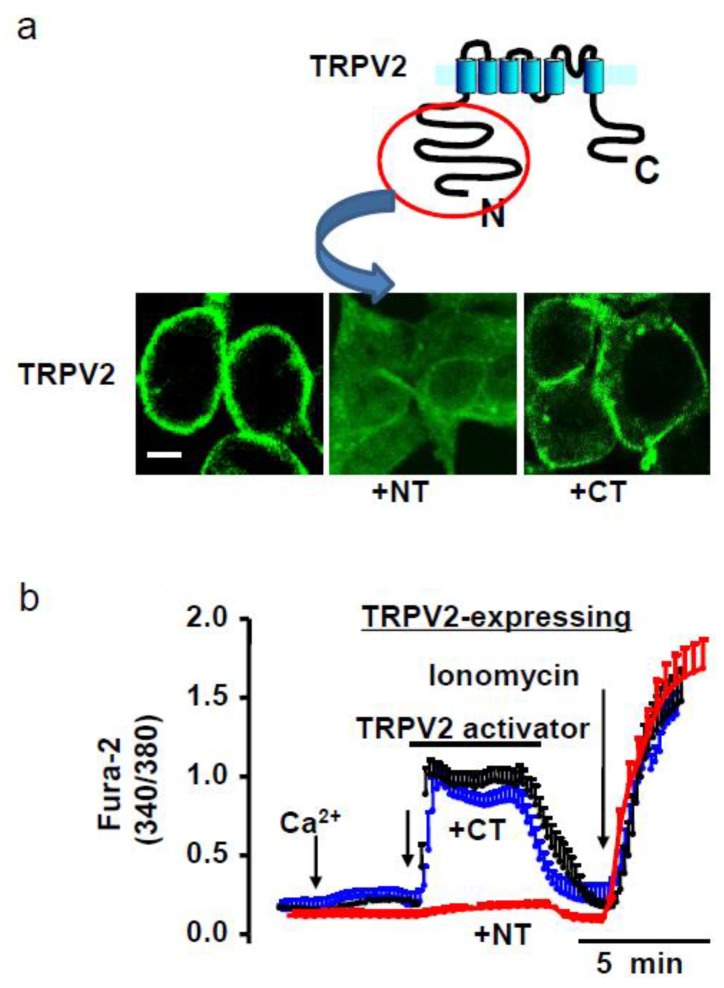
Overexpression of the TRPV2-NT domain blocks the surface expression of TRPV2. (**a**) HEK293 cells expressing TRPV2 were transfected with the amino terminal (NT) (amino acid (aa) 1-387) domain protein or the carboxy terminal (CT) (aa 633-756) protein and stained with an anti-TRPV2 antibody. Overexpression of the NT domain reduced TRPV2 surface expression. Scale bar = 10 μm. (**b**) Intracellular Ca^2+^ increase in response to extracellular Ca^2+^ (5 mM) and 2-APB (500 μM) in cell loaded with Fura-2. Overexpression of the NT domain reduced the Ca^2+^ increase [9].

To determine how to suppress the accumulation of TRPV2 in the plasma membrane, we found that a part of the amino terminal (NT) domain of TRPV2 protein plays a role in retaining TRPV2 on the cell surface. Therefore, the NT domain protein could suppress the plasma membrane accumulation of TRPV2 (Figure 2). We recently reported that reducing the accumulation of TRPV2 in the sarcolemma by overexpressing its NT domain protein prevented cardiac dysfunction [9,17] and DCM progression in animal models while also enhancing survival [9].

### 4.2. Dominant-Negative Strategy

We developed procedures to inhibit endogenous TRPV2 activity, such as the skeletal muscle-specific Tg incorporation of dominant-negative (DN)-*TRPV2*. This activity was assessed using a TRPV2 mutant model with a mutation in the putative pore region that was produced either by crossing DN-*TRPV2* Tg mice with *mdx*, or by infecting BIO14.6 hamsters with an adenovirus carrying the DN-*TRPV2* gene. We reported that expression of DN-*TRPV2* ameliorates muscle pathology in *mdx* mice and J2N-k hamsters by preventing abnormal Ca^2+^ signaling [18]. Furthermore, inhibition of endogenous TRPV2 in *mdx* skeletal muscles was largely protected from eccentric contraction-induced force loss [19].

### 4.3. Functional Antibody and Tranilast

We recently succeeded in creating neutralizing antibodies that inhibit TRPV2 activity from the extracellular side [20]. We tested the therapeutic efficacy of TRPV2 inhibition in J2N-k hamsters and found that abnormal Ca^2+^ influx and stretch-induced muscle damage in J2N-k myocytes were significantly reduced by these antibodies. Further, we observed that the treatment with one of the antibodies ameliorated the severity of cardiac dysfunction as well as muscular dystrophy evaluated by echocardiography and tissue with Masson’s trichrome staining [21,22]. Administration of this antibody improved heart failure pathologies in the murine heart failure of the 4C30 DCM model or the TAC-induced model [21,22].

We and others previously found that tranilast, an anti-allergic drug, effectively inhibits TRPV2 channel activity [9,29,30]. Tranilast is a TRPV2 inhibitor that is commercially available to researchers as well as clinicians. We observed that tranilast reduced the accumulation of TRPV2 in the sarcolemma as well as abnormal Ca^2+^ mobilization by 2-APB stimulation in the cardiomyocytes of DCM model animals (J2N-k, 4C30; unpublished observation). Oral administration of tranilast to DCM animals (J2N-k, 4C30) reduced TRPV2 on the sarcolemma of their hearts, similar to the results observed after overexpressing the TRPV2 NT domain. Tranilast markedly reduced ventricular dilation and muscle fibrosis in J2N-k hearts [9]. Furthermore, it improved cardiac contraction in the same animals, as evidenced by a decrease in echocardiographic parameters (LVDd and LVDs) to levels seen in WT animals, as well as an improvement in fractional shortening (FS) [9]. Similar beneficial effects of tranilast were observed in the 4C30 DCM model even with end-stage heart failure (FS < 10%) as well as early-stage [26,27].

Although skeletal muscle pathogenesis associated with DMD has been extensively characterized, the cardiomyopathy and progression to heart failure have not been well studied. Dystrophin-deficient *mdx* mice only develop mild indicators of cardiomyopathy in the early stage of the disease. However, it is reported that *mdx* mice additionally lacking utrophin (*utrn^−/−^; mdx*) (*DKO*) show severely reduced cardiac contractile function and histological indicators of cardiomyopathy between 8 and 10 weeks of age [31].

Lorin et al. reported abnormal Ca^2+^ signals in *mdx* cardiomyocytes during hypoosmotic stress. TRPV2 was found to be involved in the abnormal Ca^2+^ signals with the use of tranilast, a pore-blocking antibody, and ablation of TRPV2 by small-interfering RNA [23]. Aguettaz et al. used the cardiac myocytes isolated from *mdx* mice between 10 and 12 months of age to evaluate the localization and the pathological function of TRPV2. They used an antibody raised against an extracellular epitope of the TRPV2 and tranilast to inhibit TRPV2 activity. This report showed that sarcolemmal TRPV2 plays a key role in cation influx and subsequent dysregulation in dystrophin-deficient cardiomyocytes and enhanced under stretching conditions [24,25].

**Figure 3:**
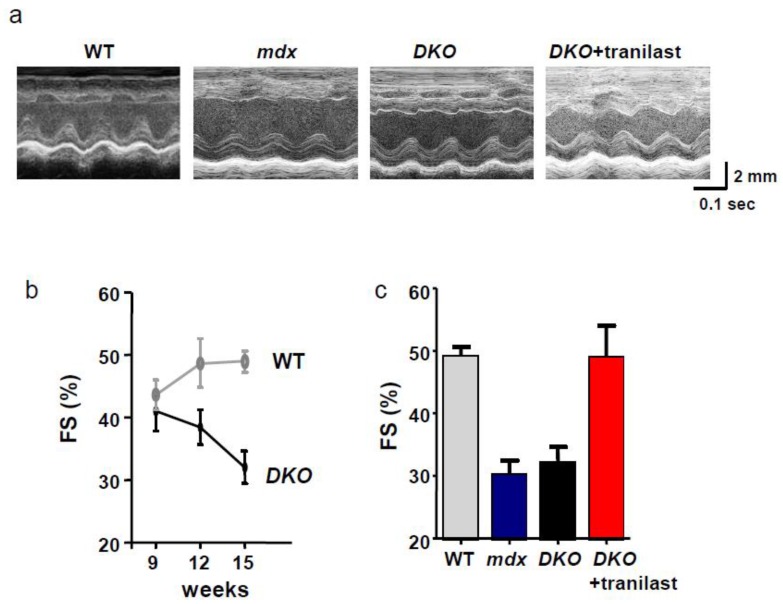
Characterization of the cardiac function in dystrophin-deficient (*mdx*) mice and dystrophin/utrophin double-knockout (*DKO*) mice. (**a**) Representative M mode echocardiograms of each group of mice at 15 weeks of age. (**b**) Age-dependent changes in cardiac function measured as fractional shortening (FS), and (**c**) FS at 15 weeks of age. Cardiac function decreased by about 20% (especially in the myocardial anterior) in *mdx* and *DKO* mice at 15 weeks of age. The decrease in cardiac function seen in *DKO* mice was suppressed by the administration of tranilast. Data of *mdx* at 15 weeks of age was shown for comparison.

We measured the echocardiography of the *DKO* mice and compared the readings with those from WT mice. The FS values of *DKO* and WT were almost similar at 9 weeks of age. Afterwards, the FS value of *DKO* mice was decreased gradually, whereas the FS value of WT mice was slightly increased. The FS value of *DKO* mice at 15 weeks of age was not so severe and almost the same as that of age-matched mdx mice. When tranilast was administered to 5-week-old *DKO* mice for 10 weeks, cardiac dysfunction, especially within the anterior wall of the heart, was prevented at 15 weeks of age (Figure 3). Although we detected the beneficial effects of tranilast on cardiac dysfunction in *DKO* mice, we need to develop the more severe animal models that mimic the symptoms of cardiomyopathy seen in DMD.

### 4.4. Gene Deletion

Studies using *TRPV2*-deficient animals have also confirmed the pathological significance of TRPV2. The cardiac hypertrophy and/or the cardiac dysfunction observed in TAC and/or MI rodent models were suppressed in global *TRPV2* KO mice [12,14].

## 5. Development of TRPV2 Inhibitors

Although TRPV2 seems to be an excellent therapeutic target for cardiomyopathy/heart failure and MD, no specific inhibitors have been identified until recently. The lack of a reproducible TRPV2 activity assay system may be one of the reasons for this failure to identify TRPV2 inhibitors. Cannabidiol (CBD), probenecid, lysophosphatidylcholine (LPC), 2-APB, high temperature (> 52 °C), and stretch are known to activate TRPV2. However, these stimulations did not always produce a sufficient response nor reproducible results for the inhibitor assay [28]. We found that mouse TRPV2 activity could be reproducibly evaluated by 2-APB stimulation under acidic conditions (pH = 6.5). Using the above method, we found that SKF96369 and tranilast inhibit Ca^2+^ influx through mouse TRPV2 [28]. Subsequently, tranilast and SKF96369 were used as lead compounds, and the novel compounds that inhibit TRPV2 channel activity at a lower concentration than tranilast were identified using an in silico search for analogous compounds based on interaction prediction and an inhibitor search using commercially available compounds. These novel compounds, including lumin (NK-4), inhibited TRPV2 activity and yielded a reduction in fibrosis and improved cardiac function in J2N-k hamsters [28]. 

Recently, Chai et al. identified three putative functional ligand binding sites for the TRPV2 channel using a protocol based on structural and evolutionary information. This investigation also determined potential inhibitors that would target these sites. They optimized the best hit and developed SET2 as a potent TRPV2-selective antagonist against 2-APB (IC_50_ = 0.46 μM), SET2; *N*-(Furan-2-ylmethyl)-3-((4-(*N*7-methyl-*N*’-propylamino)-6-(trifluoromethyl)-pyrimidin-2-yl) thio)-propanamide [32]. Schiano et al., searching for structurally related synthetic or endogenous lipids with capsaicin derivatives, identified olvanil (C18:1) and compound **73** (18:1) as potent TRPV2 antagonists against LPC stimulation (0.16 and 0.12 μM, respectively) and linoleoyl ethanolamide as a potent TRPV2 antagonist against CBD stimulation (0.65 μM) [33].

## 6. Clinical Trials of TRPV2 Inhibitors

**Figure 4:**
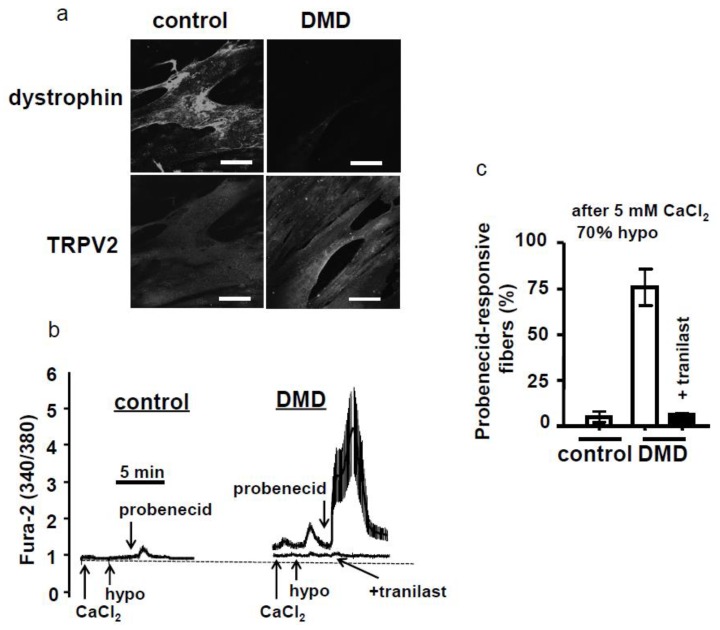
Characterization of the control and human Duchenne muscular dystrophy (DMD) myotubes and effects of tranilast on the DMD myotubes. (**a**) Human control myotubes (KD3) and dystrophic myotubes (D4P4) produced from human DMD patients were visualized by immunofluorescence using an anti-TRPV2 or anti-dystrophin antibody. Scale bar = 50 μm. (**b**) Representative traces for the intracellular Ca^2+^ response. Myotubes placed in a solution containing 2 mM CaCl_2_ were stimulated with high Ca^2+^ (5 mM CaCl_2_, indicated by arrow) and then a 70% hypoosmotic medium (hypo, indicated by arrow). Myotubes were further perfused with the medium containing 38 μM probenecid (probenecid, indicated by arrows). In one experiment, 100 μM tranilast was included in the perfusion medium. (**c**) The ΔF-ratio was calculated by subtracting the resting fluorescence ratio from the maximal ratio after the inclusion of probenecid. Myotubes exhibiting a ΔF-ratio > 0.3 were defined as probenecid-responsive myotubes. Only DMD myotubes were responsive to probenecid, which was blocked completely by tranilast [34].

It is necessary to develop human-specific inhibitory antibodies or inhibitors of TRPV2 for treating human patients. Before finding adequate inhibitors, we characterized the DMD myotubes and tested tranilast on them [34], because we previously found tranilast can block hTRPV2 [28]. In this study, the human control skeletal muscle cell line, KD3 (control), was compared with the DMD cell line, D4P4 (DMD), from DMD patients. Although dystrophin was detected in the sarcolemma of control myotubes, it was not detected in DMD myotubes (Figure 4a). Whereas TRPV2 was highly localized in the sarcolemma DMD myotubes, membrane accumulation of TRPV2 was almost nonexistent in control myotubes (Figure 4a). We measured the change in [Ca^2+^]_i_ in these myotubes. Since human TRPV2 is not activated by stimulation with 2-APB [35], we used probenecid as an activator following stimulation of myotubes with high Ca^2+^ and a hypoosmotic environment. When DMD myotubes were perfused with high Ca^2+^-containing medium and then 70% hypoosmotic medium, there was a slight increase in [Ca^2+^]_i_ (Figure 4b). Although probenecid treatment massively increased [Ca^2+^]_i_, this increase was almost completely abolished by treatment with tranilast (Figure 4b,c). These results suggested that tranilast has therapeutic potential for treating DMD.

While we already mentioned the effect of tranilast on sarcolemma TRPV2 in the cardiomyopathic hearts in Section 4.3, tranilast has long been reported to be effective in treating heart failure [36,37,38], for example also canine heart failure [38]. In these cases, its mechanism of action is considered to prevent degranulation of mast cells [36,37,38] within the cardiac muscles. Since mast cell numbers are thought to increase during heart failure [37], blockade of the degranulation is believed to prevent deterioration of cardiac function. Since the degranulation is caused by TRPV2 activation [39], it is thought that activation of TRPV2 in the mast cells within the cardiac muscles as well as cardiac muscles themselves would play an important role in the progression and/or deterioration of heart failure. Huang et al. reported that tranilast directly binds to Nod-like receptor pyrin domain containing 3 (NLRP3) to inhibit the activation of the NLRP3 inflammasome [40], thus preventing the release of proinflammatory cytokines. Tranilast seems to have therapeutic effects on diseases characterized by abnormal activation of the NLRP3 inflammasome as well as abnormal activation of TRPV2. However, tranilast has not yet been used clinically for treating cardiomyopathy and/or heart failure.

We performed a pilot study [41] to investigate the effect and safety of tranilast in patients with advanced heart failure. The participants were two patients with MD. The first case (P1) was a 45-year-old man with BMD, and the second case (P2) was a 46-year-old man with Emery–Dreifuss MD. Both patients were resuscitated from cardiopulmonary arrest and suffered from severe heart failure despite maximum therapy. In both patients, serum brain natriuretic peptide (BNP) levels were >100 pg/mL, and FS was only 4% in P1 and 6% in P2. After obtaining informed consent, we prescribed a 3 × 100 mg/day tranilast treatment regimen for both patients.

**Figure 5:**
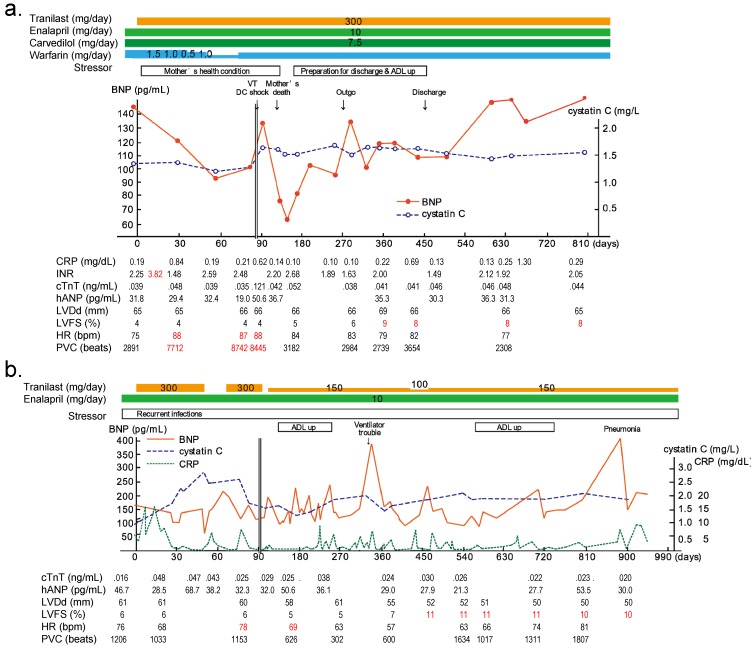
Clinical charts of the patients. (**a**) Clinical chart of the first case (P1), and (**b**) clinical chart of the second case (P2) (The data of 0−90 days were from [41]).

P1 showed a decrease in serum BNP after starting tranilast therapy, but he needed to adjust his warfarin dosage. When his mother’s health condition deteriorated, his heart rate increased and premature ventricular beats became more frequent. However, these phenomena were settled down after the mother’s death. After one year of the therapy, FS increased to 8% and the patient was discharged from our hospital [42] (Figure 5a). After discharge, BNP returned to the pre-treatment level due to increased activity, whereas FS remained stable over one year.

P2 showed recurrent multi-organ infections, and his serum BNP levels were unstable. Because cystatin C was elevated after starting tranilast, we stopped administering tranilast 52 days after initiating treatment. After stopping treatment, BNP increased from 64.5 to 228.5 pg/mL within two weeks. After tranilast treatment resumed, the patient’s BNP decreased to 114.6 pg/mL. With concern over the adverse effects of tranilast on renal function, we limited the dose of tranilast to 150 mg/day. LVDd was decreased and FS was increased after 1.5 years of treatment [42] (Figure 5b).

According to the levels of expression of TRPV2 in circulating mononuclear cells (MNCs), TRPV2 was diffusely expressed in the cytoplasm of cells from control samples. In contrast, TRPV2 was preferentially localized at the plasma membrane in both patients. After initiation of tranilast, the percentage of MNCs with strong expression of TRPV2 at the plasma membrane was decreased in both patients [41].

These facts suggested that tranilast inhibits TRPV2 in humans and is effective for treating cardiomyopathy associated with muscular dystrophy.

At present, advanced medicine B is promoting “a multicenter joint non-blind single-group study of TRPV2 inhibitors for myocardial injury in muscular dystrophy” for the repositioning of tranilast for human cardiomyopathy.

## 7. Conclusions

It was revealed that plasma membrane expression of TRPV2 is enhanced and activated in cardiomyopathy, and TRPV2 inhibition decreases plasma membrane localization of TRPV2, abnormal Ca^2+^ signaling, and muscle degeneration. Tranilast and several other inhibitors that inhibit TRPV2 channel activity at lower concentrations than tranilast were identified. These TRPV2 inhibitors were found to have a protective effect against DCM. In the future, the clinical application of tranilast as a TRPV2 inhibitor will be examined for cardiomyopathic patients, and further development of more selective and potent TRPV2 inhibitors for clinicians is expected.

“TRPV2 inhibition therapy” as mentioned here is expected to open up a new therapeutic strategy for muscle dysgeneses, such as cardiomyopathy/heart failure and MD.

## Figures and Tables

**Table 1:** Effects of TRPV2 blockade on muscular dystrophy and cardiomyopathy.

TRPV2 Blocker Gene, Agents	Animal Models/Cells for Human Disease	Strain	Background of Animals	Evaluation Index (Efficacy)	Reports
**TRPV2-NT Transgenic or** **adenovirus**	* DCM mice	4C30	sialyltransferase transgenic	cardiac function↑, heart weight↓,	[9]
				fibrosis↓, survival↑ * CK↓,* ANP↓, * cTn-I↓ in serum	
	DCM mice	* TNNT2 *ΔK210	cTn-T mutant knock-in	cardiac function↑	[17]
	DCM hamsters	J2N-k	δ-sarcoglycan defect	cardiac function↑	[9]
	* DOX induced CM	BL6	cardiotoxicity	cardiac function↑, *ROS production↓	[9]
* DN-*TRPV2* **Transgenic** **or adenovirus**	* MD mice	*mdx*	dystrophin defect	fibrosis↓, serum CK↓, Ca^2+^ influx↓, recovery of muscle strength↑	[18,19]
	MD hamsters	BIO14.6	δ-sarcoglycan defect	fibrosis↓, serum CK↓, Ca^2+^ influx↓	[18]
**Functional antibody**	DCM mice	4C30	sialyltransferase transgenic	cardiac function↑	[20,21,22]
	DCM hamsters	J2N-k	δ-sarcoglycan defect	cardiac function↑, serum CK↓	
	MD cardiomyocytes	*mdx*	dystrophin defect	stretch induced Ca^2+^ influx↓ in isolated cardiomyocytes	[23,24,25]
	TAC mice	BL6	hemodynamic stress	cardiac function↑	[22]
**Inhibitors** tranilast, * lumin	DCM mice	4C30	sialyltransferase transgenic	cardiac function↑, fibrosis↓	[26,27]
	DCM hamsters	J2N-k	δ-sarcoglycan defect	cardiac function↑, fibrosis↓	[9,28]
	MD cardiomyocytes	*mdx*	dystrophin defect	stretch induced Ca^2+^ influx↓	[23,24,25]
	MD mice	* *DKO*	utrophin/dystrophin defect	cardiac function↑	Figure 3
**Gene silencing**	MD cardiomyocytes	*mdx*	dystrophin defect	Ca^2+^ influx↓	[23]
**antisense DNA,** * **siRNA**	MD myotubes	BIO14.6	δ-sarcoglycan defect	Ca^2+^ influx↓	[8]
**Gene deletion**	* TAC mice	BL6/129S	hemodynamic stress	hypertrophy↓, cardiac function→	[12]
**(Knockout)**	* MI mice	BL6/129S	ischemia stress	cardiac function↑	[13,14]

* DCM, dilated cardiomyopathy; * MD, muscular dystrophy; * CK, creatine phosphokinase; * ANP, atrial natriuretic peptide; * cTn-I, cardiac troponin-I; * TNNT2, cardiac troponin-T; * Δ210K, 210Lys deletion; * DOX, doxorubicin; * ROS, reactive oxygen species; * DN, dominant negative; * lumin (NK-4), 4,4′-[3-[2-[1-ethyl-4(1H)-quinolinylidene] ethylidene]-1-propene-1,3-diyl]bis(1-ethylquinolinium) diiodide; * *DKO*, double knockout mouse; * siRNA, small interfering RNA; * TAC, transverse aortic constriction; * MI, myocardial infarction; ↑, increased or improved; ↓, decreased or ameliorated; →, no change.
